# The Impact of Postoperative Complications on Survivals After Esophagectomy for Esophageal Cancer: Erratum

**DOI:** 10.1097/01.md.0000471826.95786.ab

**Published:** 2015-10-02

In the article “The Impact of Postoperative Complications on Survivals After Esophagectomy for Esophageal Cancer”,^1^ which appeared in Volume 94, Issue 33 of *Medicine*, the *P* values in Tables 3 and 4 were presented incorrectly. In addition, the values in Figure 2B were originally incorrect, and this panel has also been replaced. The article has since been corrected online.
